# Ultrastructural evidence for the activation of autophagy and analysis of the protective role of autophagy in goat spermatozoa under liquid storage

**DOI:** 10.3389/fvets.2025.1543459

**Published:** 2025-03-13

**Authors:** Tengfei Liu, Jincong Niu, Yuqi Huang, Hong Chen, Yongjie Wu, Yongping Xu

**Affiliations:** ^1^College of Veterinary Medicine, Northwest A&F University, Yangling, China; ^2^College of Life Sciences, Northwest A&F University, Yangling, China

**Keywords:** goat spermatozoa, liquid storage, autophagy, ultrastructure, chloroquine

## Abstract

Liquid storage of semen is a widely used technology for promoting genetic improvement in goat breeding. The short shelf life of spermatozoa greatly limits the application of liquid storage, which urgently needs to explore the underlying regulatory factors. Autophagy as a cellular catabolic process plays critical roles in eliminating damaged material, that thus protects the function and fertilizing ability of spermatozoa. Nevertheless, the regulatory mechanisms of autophagy in goat spermatozoa under liquid storage remain unclear. In this study, the typical morphologic abnormalities and ultrastructural changes in goat spermatozoa, such as plasma membrane swollen and shrunken, acrosome exfoliation, and axoneme exposure, were observed after liquid storage at 4°C. Moreover, assessment of the formation of autophagy in liquid-stored goat spermatozoa was performed by a morphological “gold standard” of electron microscopy. Notably, a large number of vesicles with double-membrane structure indicating autophagosome were found to surround the aberrant spermatozoa, suggesting the activation of autophagy. Several proteins, such as LC3, ATG5, and p62, exhibited differential expression after liquid storage, which further validated the occurrence of autophagy in liquid-stored goat spermatozoa. Furthermore, chloroquine treatment was used to inhibit the autophagy of spermatozoa, which caused a significantly decrease in the quality of liquid-stored spermatozoa, including motility, viability, plasma membrane integrity, and acrosome integrity. Significant increase in ROS and MDA levels of spermatozoa and significant decrease in Ca^2+^ influx and protein tyrosine phosphorylation of spermatozoa were also detected after chloroquine-induced autophagy inhibition. The ultrastructural observation of double-membrane autophagosome provides strong evidences for the activation of autophagy in goat spermatozoa under liquid storage. The inhibition of autophagy mediated by chloroquine indicated that autophagy plays vital roles in the survival of spermatozoa. These results facilitate understanding the activation of autophagy in spermatozoa and provide valuable references for uncovering the underlying regulatory mechanisms of liquid storage of goat spermatozoa.

## 1 Introduction

In goat breeding, artificial insemination (AI) is one of the most important techniques to promote genetic improvement (1, 2). The efficiency and success of AI are mainly determined by the quality of spermatozoa, which requires high-standard processing and storage of spermatozoa (3, 4). Currently, there are two primary preservation strategies for goat semen: liquid storage (short-term) and cryopreservation (long-term). Liquid semen storage has prominent advantages than cryopreservation as the absence of mechanical forces, cold shock, and extreme high dilution, and obviously reduces spermatozoon damage (3, 5). Moreover, the conditions of liquid storage in easier handling and transport and higher utilization of spermatozoa are responsible for the more convenient and economic benefits (5, 6). Nevertheless, the application of liquid storage still suffers the limited shelf life and usage potential of spermatozoa that is evaluated by multiple quality indexes of spermatozoa, such as the motility, viability, and fertility of spermatozoa (6, 7). Therefore, it is meaningful to investigate the underlying molecular mechanisms of liquid storage of spermatozoa for technical improvement in goat production industry.

A series of physiological events and genetic mechanisms are involved in the fate of spermatozoa (8). Autophagy is an evolutionarily conserved cellular catabolic process facilitating the maintenance of intracellular homeostasis through lysosome-mediated degradation of cytoplasmic components (9, 10). Once autophagy is activated, the dysfunctional cytoplasmic components, such as damaged organelles and redundant proteins, are engulfed by a double-membrane autophagy vesicle and then subjected to lysosomes for degradation (10, 11). The process of autophagy/macroautophagy contains a set of consecutive steps and is initiated from the formation of an isolated membrane termed phagophore, which further matures into a double-membrane autophagosome (12, 13). The outer membrane of autophagosome subsequently fuses with the lysosome to form a degradative structure, namely autolysosome, triggering degradation (12, 13). Considerable ultrastructural investigations have approved that the occurrence of double-membrane autophagosome is a credible standard to assess the formation of autophagy (14). Moreover, many autophagy-related proteins (ATG) participating in the formation and regulation of autophagy have been widely used as specific autophagy markers (10, 13, 15). Microtubule-associated protein light chain 3 (LC3) exists in two forms of LC3-I and LC3-II and is tightly associated with the stage of membrane elongation and formation of autophagosome (16, 17). The activity of autophagy promotes the LC3-I processed to LC3-II, which specifically resides in autophagic membrane structures, and the conversion of LC3-I to LC3-II serves as an important indicator of autophagy levels (16–18). ATG5 is one of autophagy core proteins and plays essential roles in regulating autophagy through conjugating ATG12 forming autophagy machinery and then accelerating the processing of LC3-II (10, 19, 20). In addition, sequestosome-1 (SQSTM1) protein, also known as p62, a selective autophagy substrate protein, is degraded by autophagy and also used to evaluate the autophagic flux (13, 21). The function of p62 protein as an autophagy receptor is to interact with LC3 mediating autophagic degradation pathway and important for cell survival (21, 22).

Increasing studies have revealed that autophagy plays fundamental roles in various aspects of male reproductive system, such as spermatogenesis, testosterone production, and fertilization (23–25). Spermatogenesis damage was reported to be manipulated by autophagy, which is required for spermatozoa flagella biogenesis and acrosome biogenesis (26–28). In cryptorchid testis, deficient autophagy contributed to generate abnormal spermatozoa resulting in male infertility (29, 30). Germ cell-specific ATG5 and ATG7 knockout mouse caused severely infertility associated with the reduced numbers and abnormal morphology of spermatozoa (31, 32). The involvement of autophagy in normal male reproduction may imply the importance of autophagy to spermatozoa (8, 25). The autophagy functions as an adaptation mechanism against adverse conditions and external stresses by eliminating damage material and providing energy sources to overcome stress and protect the function and fertilizing ability of spermatozoa (8, 9, 33). Liquid-stored spermatozoa are exposed to a number of stresses, such as starvation, cold stress, and oxidative stress, which significantly induce autophagy (34–37). Previous studies showed that functional activity and expression change of autophagy-related proteins regulated the motility and survival of human spermatozoa (38). Exposure to oxidative stress, autophagy was activated in human spermatozoa, but the inhibited autophagy impaired the quality of spermatozoa and caused an increase in cell death (33). Although morphological and molecular evidences have demonstrated the association of autophagy with the regulation of survival of spermatozoa, few studies focus on the underlying roles of autophagy in long-term liquid storage of goat spermatozoa.

Therefore, in this study, to understand the occurrence of autophagy in goat spermatozoa, the morphological characteristics and ultrastructural changes of spermatozoa were observed under liquid storage at 4°C, which provides valuable reference for exploring the autophagy of spermatozoa on the morphological level. The expression of autophagy-related proteins was also analyzed to validate the autophagy in liquid-stored goat spermatozoa. Furthermore, chloroquine treatment was used to suppress the autophagy of goat spermatozoa, and to investigate the roles of autophagy in the survival of spermatozoa and fertility under liquid storage. These results provide insight into the roles of autophagy in regulating liquid storage of goat spermatozoa.

## 2 Materials and methods

### 2.1 Animals, semen collection and processing

All experimental procedures in this study complied with the experimental animal management regulations and ethical requirements and were approved by the Animal Care and Use Committee of Northwest A&F University (No. DY2023033), Shaanxi, China. Mature Guanzhong dairy goats (*Capra hircus*) with ages between 2 and 3 years were reared in the experimental animal center of Northwest A&F University. All the test goats were fed and managed continually according to the management standards of dairy goats. Using artificial vagina, the semen was collected from six healthy and matured sexually goats at July–October. The collected semen samples were immediately transported to laboratory condition at 37°C within 30 min. The motility and concentration of spermatozoa was evaluated using a phase-contrast microscope and calibrated spectrophotometer, and the semen samples with ≥90% motility and ≥3 × 10^9^ spermatozoa/mL concentration were pooled and used for subsequent experiments. The pooled semen samples were extended with Tris-egg yolk extender (Tris 3.63 g/100 mL, fructose 0.50 g/100 mL, citric acid 1.99 g/100 mL, egg yolk 10 mL/100 mL, penicillin 5,000 IU/100 mL, and streptomycin 0.1 g/100 mL) to a concentration of 3 × 10^8^ spermatozoa/mL. The semen samples were stored in the refrigerator at 4°C and evaluated at three different incubation times (0, 48, and 96 h) according to a previous report (39). For the experiment of autophagy inhibition, the extended spermatozoa were incubated with 0.5 mM chloroquine for 2 h at 37°C before refrigeration. The control group was incubated only with PBS solution with the same concentration of vehicle (DMSO). For the assessment of chloroquine treatment effect, protein expression analysis was performed at 0 h refrigeration. All experiments were done with at least three replicates.

### 2.2 Scanning electron microscopy analysis

The semen samples were washed twice using PBS solution after centrifuging at 500 rpm for 10 min. The pellets of spermatozoa were smeared on cover glasses (diameter of 10 mm) and air dried naturally. Then, the cover glasses were fixed in 2.5% glutaraldehyde and 2% paraformaldehyde in 0.1 M PBS with pH 7.4 and overnight at 4°C. Next, spermatozoa were post-fixed in 1% osmium tetroxide and dehydrated with an increasing gradient of ethanol. After dried with dry ice, spermatozoa were coated with gold–palladium membranes and observed under a Nano 450 scanning electron microscope (FEI Company, OR, United States).

### 2.3 Transmission electron microscopy analysis

The semen samples were washed twice with PBS and re-suspended in a fixative solution composed of 2% glutaraldehyde in 0.2 M PBS for 48 h at 4°C. The samples were washed and post-fixed in 1% osmium tetroxide for 2 h at room temperature, then dehydrated in an ethanol gradient, and embedded in epoxy resin. Ultrathin sections were obtained and mounted on cooper grids, contrasted in lead citrate and uranyl acetate. The sections were examined with a Tecnai G2 Spirit Bio-Twin transmission electron microscope (FEI Company, OR, United States).

### 2.4 Immunofluorescence staining

The semen samples were washed and re-suspended in PBS, adjusting the concentration of 1 × 10^6^ spermatozoa/mL. The sperm suspension was spread on poly-L-lysine-coated slides and fixed with 4% paraformaldehyde for 30 min at 4°C. After permeabilized with 0.1% Triton X-100 for 20 min and blocked with 3% BSA for 1 h, the samples were incubated with antibodies anti-LC3B (Abcam, ab192890, 1:200), anti-ATG5 (Abcam, ab108327, 1:100), anti-p62 (Abcam, ab109012, 1:200), and anti-phosphotyrosine (Abcam, ab179530, 1:100) overnight at 4°C. Then, spermatozoa were incubated with secondary antibody labeled with Alexa-Fluor-647 (Abcam, ab150075, 1:1,000) or Alexa-Fluor-488 (Abcam, ab150073, 1:1,000) at room temperature for 1 h, followed by the nuclei staining of DAPI (Abcam, ab104139) for 10 min. The images were captured by a fluorescence microscope (BX51; Olympus, Tokyo, Japan).

### 2.5 Western blot analysis

Western blot analysis was performed as previously described method (39). In brief, the proteins of spermatozoa were extracted and protein concentration was calculated using BCA Protein Assay kit (Solarbio). An equal amount of 50 μg protein from each group was fractionated by 10% SDS-PAGE and transferred to PVDF membranes. Membranes were incubated with primary antibodies (anti-LC3B, Abcam, ab192890, 1:1,000; anti-ATG5, Abcam, ab108327, 1:1,000; anti-p62, Abcam, ab109012, 1:5,000; anti-phosphotyrosine, Abcam, ab179530, 1:1,000; anti-β-actin, Abcam, ab8227, 1:1,000) overnight at 4°C, and then followed by incubation with the HRP-conjugated secondary antibody (Abcam, ab6802, 1:1,000) at room temperature for 1 h. For assessment of protein tyrosine phosphorylation, the fresh spermatozoa were directly subjected to protein extraction and used as non-capacitated (NC) group. In the control and chloroquine treated groups, the calcium ionophore A23187 (1 μM) were added for the capacitation of spermatozoa, and the proteins were extracted after 30 min. Protein bands were visualized using an ECL kit (Thermo Fisher Scientific), and Quantity One Analysis software (Bio-Rad) was applied to quantify the band intensity density.

### 2.6 Assessment of the motility, viability, plasma membrane integrity, acrosome integrity, and mitochondrial membrane potential of spermatozoa

The total motility and progressive motility of spermatozoa were evaluated using the computer assisted sperm analysis (CASA) system (Hamilton Thorne Research, Beverly, MA, United States) according to the reported procedure (39). The prepared semen samples were loaded into a chamber and examined under a phase-contrast microscope (Nikon TE-2000U, Tokyo, Japan).

The viability of spermatozoa was examined using the eosin-nigrosin staining technique, where the mixtures of 10 μL of semen sample and 10 μL of 0.5% eosin-nigrosin stain were placed on a pre-warmed slide. The percentages of live (unstained) and dead (stained with red) spermatozoa were calculated.

The hypo-osmotic swelling test (HOST) was used to evaluate the functional integrity of plasma membrane of spermatozoa as described by Zou et al. (40). The percentage of positive cells with swollen flagella was recorded for each sample. A minimum of 200 spermatozoa were calculated for each assay.

The acrosome integrity of spermatozoa was analyzed using fluorescein isothiocyanate-peanut agglutinin (FITC-PNA) staining according to the method described previously (41). Briefly, 30 μL semen sample was used to prepare a slide and fixed in anhydrous methanol for 10 min at room temperature. Then, 30 μL FITC-PNA solution (100 μg/mL in PBS) was spread over each slide. The slide was incubated in dark at 37°C for 30 min and then mounted with 10 μL antifade solution. The acrosome integrity of spermatozoa was examined using fluorescence microscope (BX51; Olympus, Tokyo, Japan).

The mitochondrial membrane potential was measured using the dye 5,5′,6,6′-tetrachloro-1,1′,3,3′-tetraethylbenzimidazol carbocyanine iodide (JC-1; Abcam, ab113850) as previously described (39). Briefly, 1 × 10^6^ spermatozoa were used and stained with 10 μM JC-1 solution at 37°C for incubating 30 min, followed by observation using the flow cytometry (FACS Calibur, BD Biosciences, San Jose, United States).

### 2.7 Measurement of ATP level, reactive oxygen species, antioxidant enzyme activities, and MDA concentration

The ATP content of spermatozoa was measured using an ATP Assay Kit (S0026, Beyotime, Shanghai, China) according to the manufacturer’s instructions. The commercial assay kits (E004-1-1, A001-3, A007-1, A005; Nanjing Jiancheng Bioengineering Institute, China) were used to assess the activities of reactive oxygen species (ROS), superoxide dismutase (SOD), catalase (CAT), and glutathione peroxidase (GSH-Px) according to the manufacturer’s instructions. The levels of ROS, SOD, CAT, and GSH-Px were determined at 488 nm, 450 nm, 405 nm, and 412 nm using a microplate reader (Multiskan Spectrum, Thermo), respectively. The MDA concentration representing the lipid peroxidation of spermatozoa was determined by the thiobarbituric acid method using an MDA assay kit (A003-1, Nanjing Jiancheng Bioengineering Institute, China) according to the previous study (39). The MDA level was measured at 535 nm using a microplate reader (Multiskan Spectrum, Thermo).

### 2.8 Measurement of changes in calcium influx of spermatozoa

Measurement of calcium (Ca^2+^) influx in spermatozoa was performed as previously described (42). The semen sample was loaded with 5 μM Fluo-4 AM (Molecular Probe) for 30 min at room temperature in the dark and washed twice to remove free Fluo-4 AM. The fluorescence signals were measured by an EnSpire^®^ Multimode Plate Reader (Perkinelmer, United States). The values were recorded at the initial five reading every 1 min, and then every 5 min for a total of 60 min after adding A23187. Data were normalized using the following equation Δ*F*/*F*_0_ = (*F* − *F*_0_)/*F*_0_, where *F*_0_ is the average of the first five readings before the addition of A23187, and *F* is the fluorescence intensity obtained at each time point.

### 2.9 Statistical analysis

Data are expressed as the mean ± SD from at least three independent experiments. SPSS 20 software was used for statistical analysis. Independent samples *t*-test was used to compare the differences between two experimental groups, and one-way ANOVA was used to compare the difference among multiple groups. A *p*-value <0.05 was considered statistically significant.

## 3 Results

### 3.1 Morphology observation of goat spermatozoa during liquid storage

**Figure 1:**
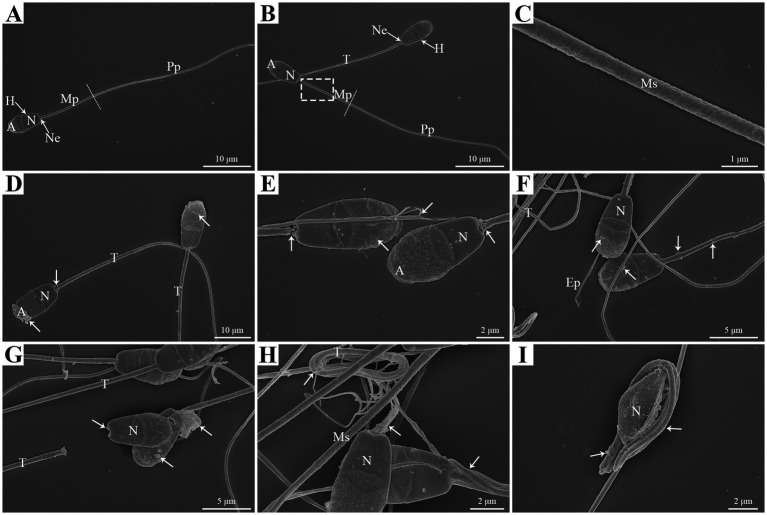
SEM analysis of morphologic changes in goat spermatozoa at 0 h **(A,B,C)**, 48 h **(D,E,F)**, and 96 h **(G,H,I)** of liquid storage. The dashed box in **(B)** was enlarged in **(C)**. H, head; Ne, neck; T, tail; Mp, midpiece; Pp, principal piece; A, acrosome; N, nucleus; Ms, mitochondrial sheath. Scale bar = 10 μm **(A,B,D)**, 5 μm **(F,G)**, 2 μm **(E,H,I)** and 1 μm **(C)**.

In this study, scanning electron microscopy (SEM) analysis was performed to investigate the damage effects of liquid storage on the structural and morphological characteristics of goat spermatozoa. The normal morphology of goat spermatozoa was observed at 0 h under liquid storage, which was characterized by the shape of head, neck, and tail regions (Figures 1A,B). Apparent acrosome and nucleus were present in the head region. The tail region contained the midpiece, principal piece, and end piece. There was a spermatozoon annulus that was considered an electron-dense ringed structure of spermatozoa that separated the principal piece from midpiece (Figure 1B). The midpiece of spermatozoa presented regularly arranged mitochondria and had the structure of mitochondrial sheath (Figure 1C).

Notably, the morphologic abnormalities of spermatozoa were observed after 48 h liquid storage, with the swollen plasma membrane covering the acrosome (Figure 1D), acrosome exfoliation (Figure 1D), neck fracture (Figure 1E), and axoneme exposure (Figure 1E). In addition, the anomalies of spermatozoa, such as the plasma membrane shrunken and the formation of vesicles in the membrane and the mitochondria were also detected (Figures 1D–F). More apparent anomalies were observed after 96 h liquid storage (Figures 1G–I). Furthermore, the aberrant structures were also detected, such as the tail twisting and coiling (Figure 1H) and the tail coiled around the head (Figure 1I).

### 3.2 Ultrastructural characteristics of goat spermatozoa during liquid storage

**Figure 2:**
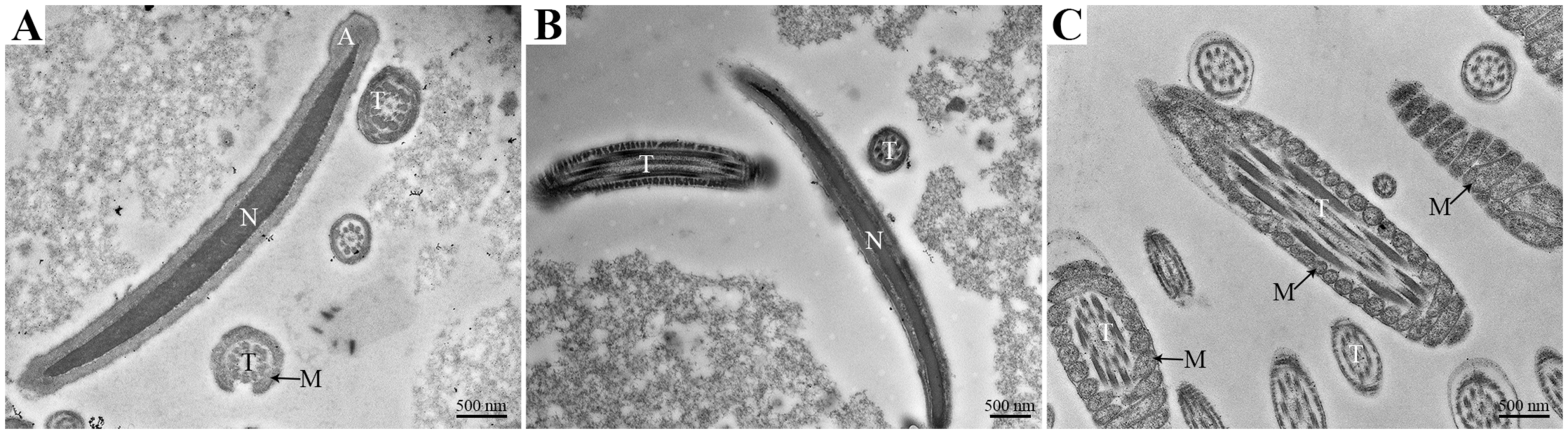
The head **(A,B)** and tail **(C)** regions of goat spermatozoa exhibit the normal ultrastructure at 0 h of liquid storage by TEM analysis. A, acrosome; N, nucleus; T, tail; M, mitochondria. Scale bar = 500 nm.

Transmission electron microscopy (TEM) analysis also observed the normal morphology of goat spermatozoa at 0 h under liquid storage. The head of spermatozoa exhibited intact acrosome, the complete and smooth surface of plasma membrane, and highly condensed nucleus with homogeneous electron-dense chromatin (Figures 2A,B). The tail of spermatozoa displayed the intact mitochondria that had intact mitochondrial sheath structure, were nearly equal in size, and regularly arranged (Figures 2B,C). The mitochondria formed gyres around the central axoneme, which was composed of some of the underlying microtubules (Figure 2C).

**Figure 3:**
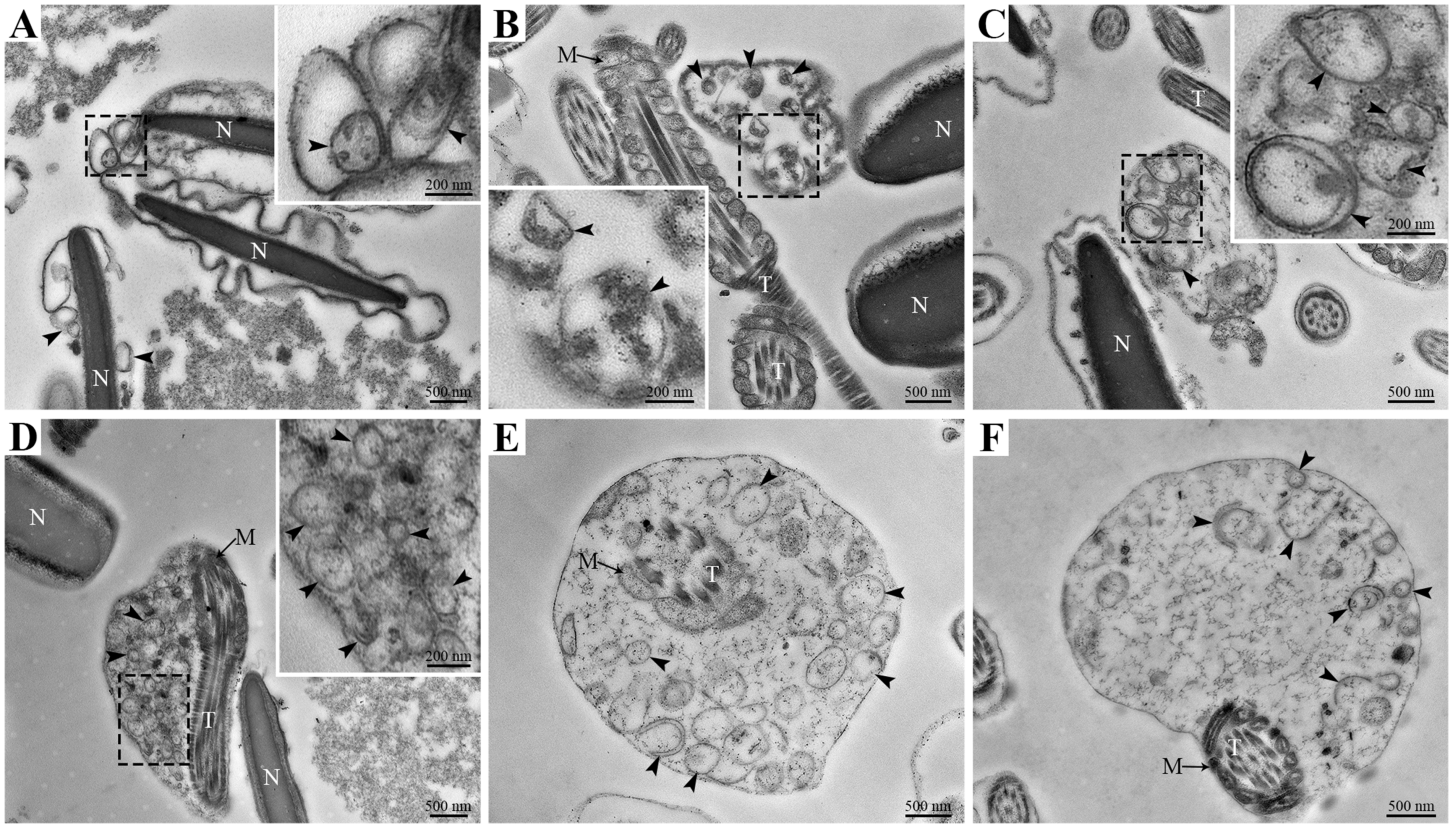
TEM analysis of ultrastructural changes in goat spermatozoa at 48 h of liquid storage. **(A–C)** The head region of spermatozoa. **(D–F)** The tail region of spermatozoa. N, nucleus; T, tail; M, mitochondria. The arrowheads mark the double-membrane autophagosome. Scale bar = 500 nm.

**Figure 4:**
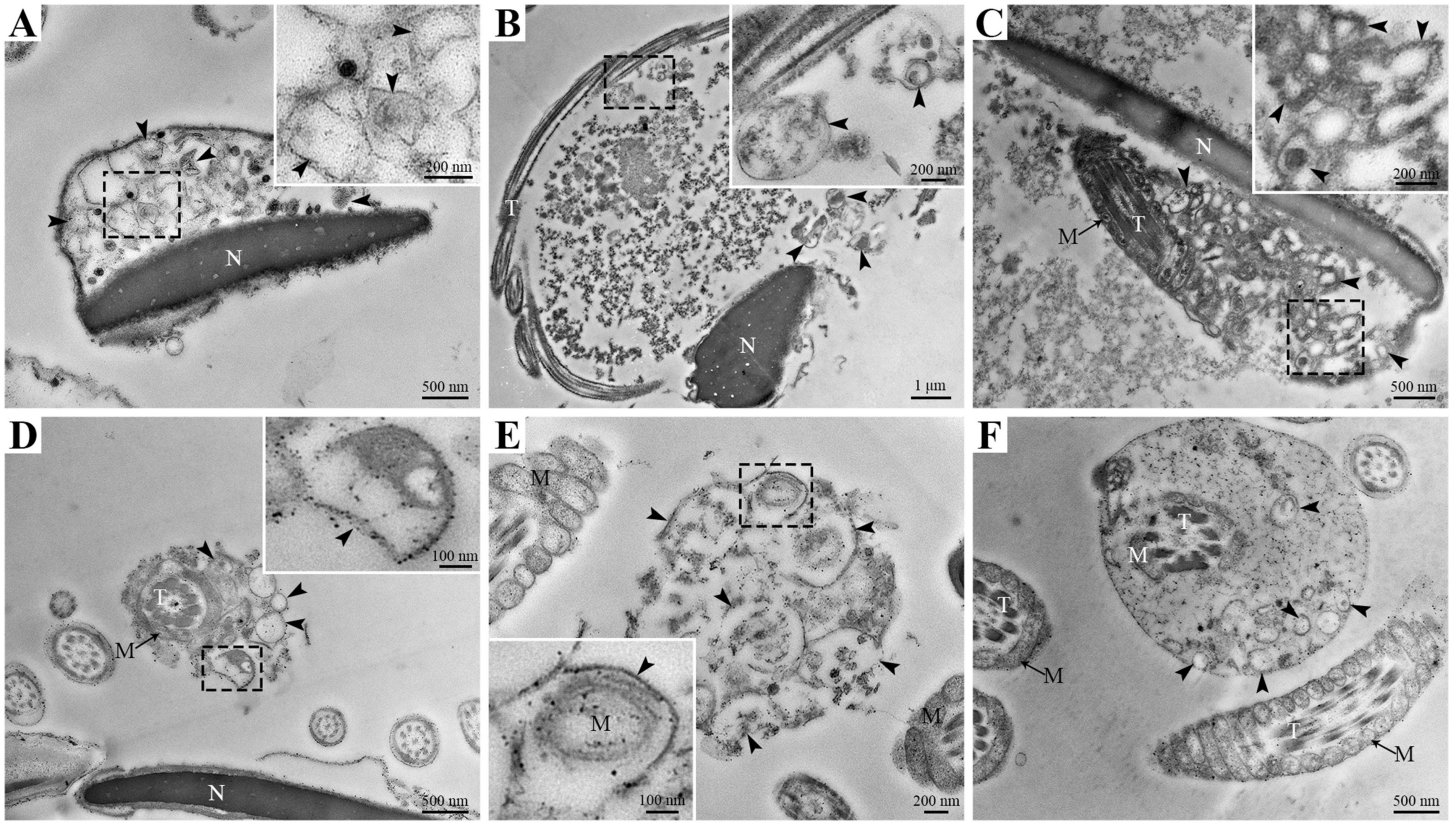
TEM analysis of ultrastructural changes in goat spermatozoa at 96 h of liquid storage. N, nucleus; T, tail; M, mitochondria. The arrowheads mark the double-membrane autophagosome. Scale bar = 1 μm **(B)**, 500 nm **(A,C,D,F)** and 200 nm **(E)**.

However, the ultrastructural changes in goat spermatozoa at 48 h under liquid storage were found by TEM. The spermatozoa presented the obvious ultrastructural abnormalities in the head and tail regions. The head was covered by the plasma membrane that swelled and appeared to emerge the membrane vesicles (Figures 3A–C). The vesicles were composed of multiple autophagosome that had obvious double-membrane structure (marked as an arrowhead) and wrapped the un-degraded substances. The tail of spermatozoa was also surrounded by the membrane vesicles that contained a large amount of double-membrane autophagosome (Figures 3D–F). Similarly, the ultrastructural damages of spermatozoa that the membrane vesicles surrounded the head and the tail and contained a large number of autophagosome were clearly visible after 96 h liquid storage (Figures 4A,D–F). In addition, the deformed spermatozoa with folded head, coiling tail and massive autophagy vesicles were observed on TEM (Figures 4B,C), which was accordant with the observation on SEM. Interestingly, the mitochondria covered by autophagosome was also detected (Figure 4E).

### 3.3 Immunolocalization and expression changes of autophagy-related proteins in goat spermatozoa during liquid storage

**Figure 5:**
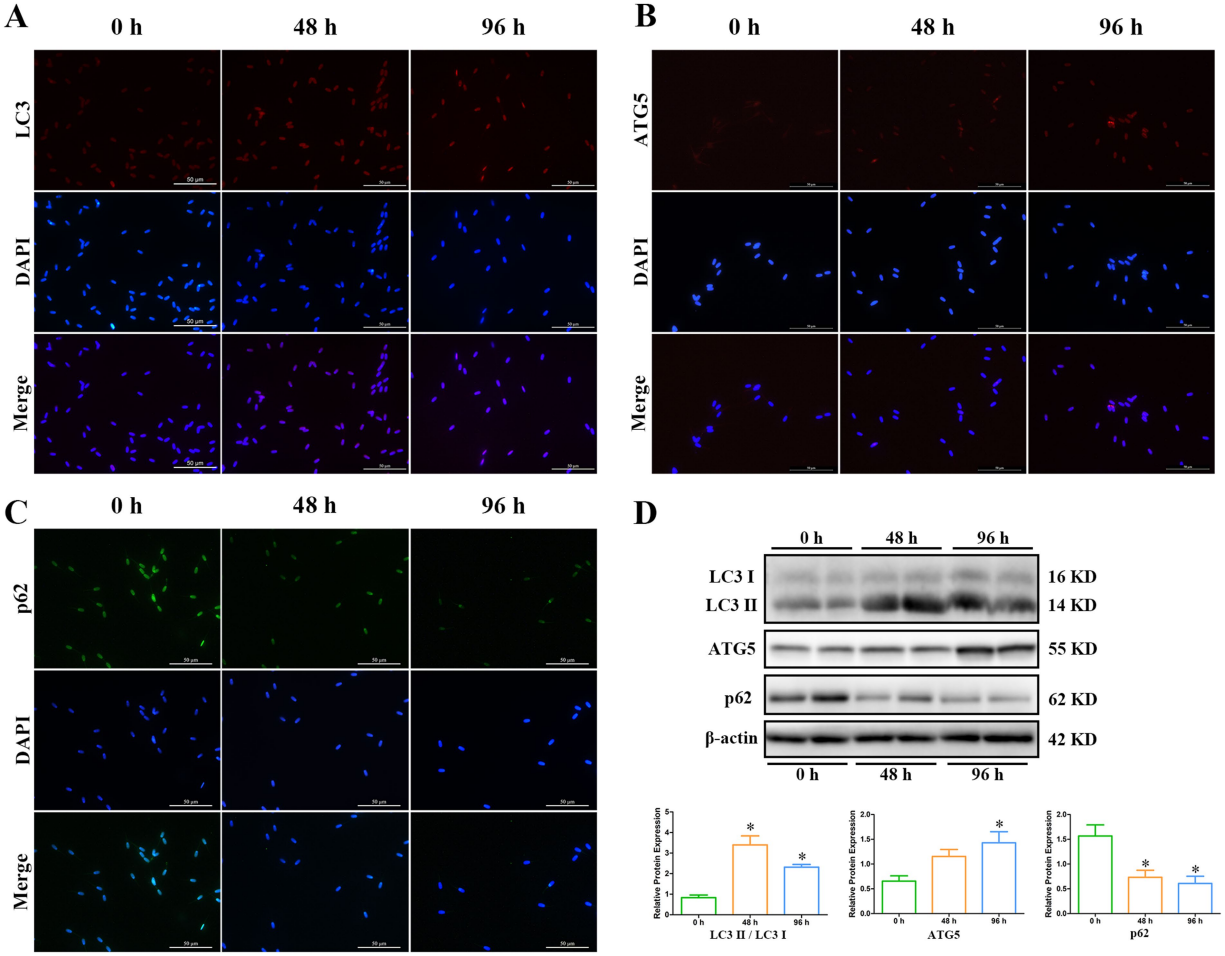
Expression analysis of LC3, ATG5, and p62 proteins during liquid storage by immunofluorescence staining **(A–C)** and western blot **(D)**. ^*^p < 0.05 compared to 0 h.

Involvement of autophagy-related proteins, such as LC3, ATG5, and p62, is essential for the formation of autophagosome (17, 20, 22). Immunofluorescence analysis revealed that the localizations of LC3 and ATG5 were mainly in the head of spermatozoa (Figures 5A,B), and p62 was detected both in the head and tail of spermatozoa (Figure 5C), which was consistent with the distribution of autophagosome on TEM analysis. The fluorescent signal of LC3 and ATG5 gradually enhanced at 48 h and 96 h, but p62 correspondingly decreased during liquid storage (Figures 5A–C). Further expression levels of LC3, ATG5, and p62 proteins were investigated by western blot analysis. The rate of LC3II/ LC3I level was significantly upregulation at 48 h and 96 h, compared to 0 h (Figure 5D). The protein level of ATG5 significantly increased at 96 h, but the level of p62 significantly decreased at 48 h and 96 h (Figure 5D). The results from western blot analysis in combination with the assessment of immunofluorescence implied the increase in autophagy level of spermatozoa, which was accordant with the observed formation of autophagy on TEM during liquid storage.

### 3.4 Effects of autophagy inhibition on the quality of goat spermatozoa during liquid storage

**Figure 6:**
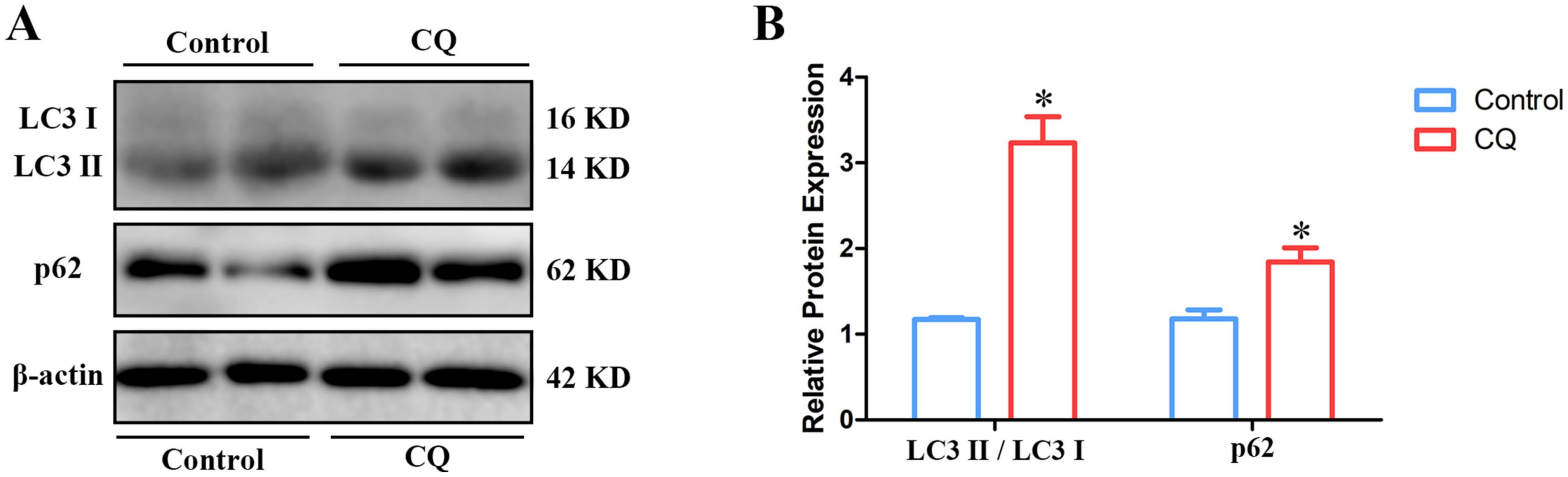
Assessment of the inhibition of autophagy in goat spermatozoa after chloroquine treatment. **(A)** Western blot analysis. **(B)** The relative protein expression based on **(A)**. CQ, chloroquine treatment. ^*^p < 0.05.

To explore the putative roles of autophagy on the liquid storage of goat spermatozoa, the inhibition of autophagy was performed using chloroquine treatment to evaluate the changes in quality parameters of spermatozoa. In this study, the inhibition effects of chloroquine treatment on autophagy were firstly assessed. After chloroquine treatment, the rate of LC3II/ LC3I level was significantly raised and the expression level of p62 protein was significantly increased compared to the control group (Figure 6), which suggested the significant suppression of autophagy.

**Figure 7:**
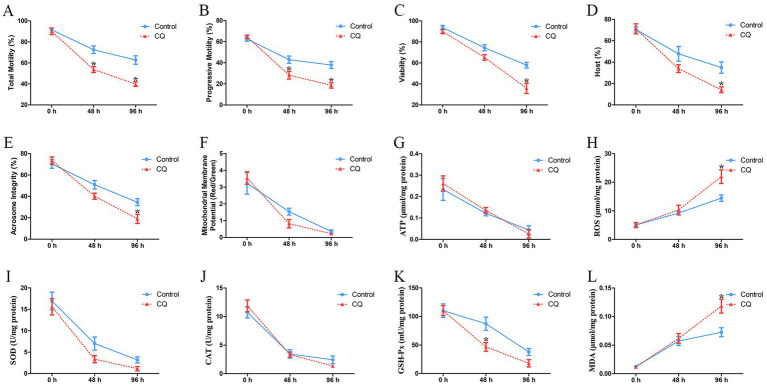
Analyses of the quality parameters of goat spermatozoa under liquid storage after chloroquine treatment. **(A)** The total motility. **(B)** The progressive motility. **(C)** The viability. **(D)** Plasma membrane integrity. **(E)** Acrosome integrity. **(F)** Mitochondrial membrane potential. **(G)** The level of ATP. **(H)** The level of ROS. **(I)** The activity of SOD. **(J)** The activity of CAT. **(K)** The activity of GSH-Px. **(L)** The level of MDA. CQ, chloroquine treatment. ^*^p < 0.05 compared to the corresponding control group.

Furthermore, the effects of chloroquine-mediated autophagy inhibition on the quality of goat spermatozoa were analyzed. The percentages of total motility and progressive motility of spermatozoa were significantly decreased both at 48 h and 96 h after chloroquine treatment, compared to the untreated control (Figures 7A,B). The viability of spermatozoa was significantly decreased at 96 h after chloroquine treatment (Figure 7C). Both the plasma membrane integrity and acrosome integrity of spermatozoa were significantly decreased at 96 h after chloroquine treatment (Figures 7D,E). There was no significant difference in the mitochondrial membrane potential and ATP level of chloroquine-treated spermatozoa compared to the untreated control, although the mitochondrial membrane potential and ATP level were gradually decreased with the liquid storage time (Figures 7F,G). The ROS level of liquid-stored spermatozoa was significantly increased at 96 h after chloroquine treatment (Figure 7H). In addition, the activities of important enzymes SOD, CAT, and GSH-Px in the antioxidant defense system were detected, and a significant decrease in the level of GSH-Px was found in chloroquine-treated spermatozoa at 48 h than that in the control group (Figures 7I–K). Moreover, the MDA level of chloroquine-treated spermatozoa was significantly increased at 96 h (Figure 7L). These results indicate that the inhibition of autophagy affected the quality of goat spermatozoa under liquid storage.

### 3.5 Effects of autophagy inhibition on Ca^2+^ influx and tyrosine phosphorylation in goat spermatozoa during liquid storage

**Figure 8:**
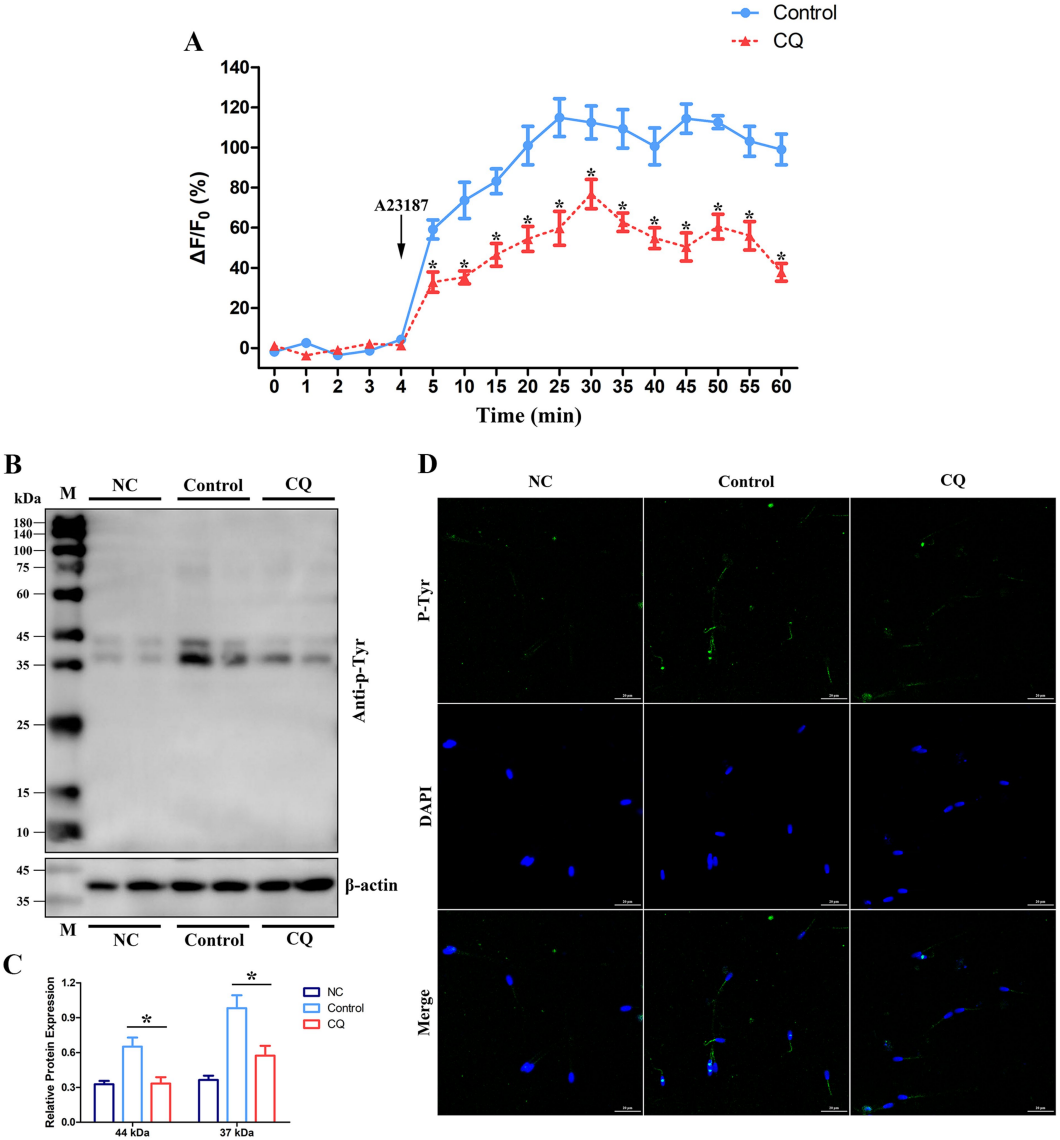
Changes in Ca^2+^ influx and protein tyrosine phosphorylation in goat spermatozoa under liquid storage after chloroquine treatment. **(A)** Ca^2+^ influx was monitored with the time-course curve of Ca^2+^ fluorescence. A23187 was added after incubation with 4 min. **(B,C)** Detection of tyrosine phosphorylation by western blotting. **(D)** Immunofluorescence of protein tyrosine phosphorylation. CQ, chloroquine treatment; M, protein marker; NC, non-capacitated spermatozoa; anti-p-Tyr, anti-phosphotyrosine. ^*^p < 0.05.

One of the important assessments for the quality of spermatozoa during liquid storage is to maintain the capacitation or fertilizing capability of spermatozoa, which is associated with changes in Ca^2+^ influx and protein tyrosine phosphorylation of spermatozoa (42, 43). In this study, changes in Ca^2+^ influx and protein tyrosine phosphorylation in goat spermatozoa were examined after chloroquine treatment under liquid storage. The analyses were performed at 96 h that exhibited the significantly decrease in the quality of spermatozoa by chloroquine-induced autophagy inhibition (Figure 7). Evaluation of Ca^2+^ influx showed that the fluorescence signals of spermatozoa were immediately increased after adding A23187 in all the groups, while chloroquine-treated spermatozoa exhibited lower levels of fluorescence than that in the control group (Figure 8A). Western blotting analysis of protein tyrosine phosphorylation showed that two bands at 44 and 37 kDa were obviously observed. The total protein tyrosine phosphorylation levels were significantly decreased at 96 h compared to the control group (Figures 8B,C). In addition, immunofluorescence analysis revealed that the fluorescent signal was obviously decreased in chloroquine-treated spermatozoa (Figure 8D), which was consistent with the results of western blotting analysis. In general, chloroquine-induced autophagy inhibition caused the decreases in Ca^2+^ influx and protein tyrosine phosphorylation of spermatozoa, which further indicated that autophagy may play positive roles in the capacitation of spermatozoa under liquid storage.

## 4 Discussion

Liquid storage is an important and widely used method for semen preservation in AI of goat breeding and offers many benefits for maintaining the fertility rates and functional capacity of spermatozoa compared with other preservation technologies (3, 44, 45). Nevertheless, the principal disadvantage of liquid-stored semen remains the short shelf life of spermatozoa (45, 46). Although some efforts have been demonstrated to increase the duration of liquid storage by optimizing the composition of extenders, liquid storage leads to a decrease in the motility, viability, and plasma membrane integrity of spermatozoa with the extended storage time (7, 47–49). Therefore, increasing studies are devoted to elucidate the physiological and molecular mechanisms regulating the survival of spermatozoa, which is undoubtedly a significant approach to improve the technologies of liquid storage and extend the lifespan of spermatozoa under liquid storage.

One of the intracellular mechanisms controlling cell death is autophagy, whose prominent role is the degradation of damaged components to maintain cytoplasmic homoeostasis and provide energy source (9, 10). Autophagy is physiologically activated by stressful conditions and extrinsic stimuli and functions as a defense mechanism to promote cell survival (9, 50, 51). Many adverse factors, such as oxidative stress and starvation, have been well-known to cause irreversible injuries to the structures and functions of spermatozoa during storage (3, 33, 46). Extensive reports have revealed a significant decrease in motility of goat spermatozoa during liquid storage (7, 46, 49). Our previous study also showed a time-dependent decline in the motility of spermatozoa and found mitochondrial damage in liquid-stored goat spermatozoa (39). In this study, SEM analysis observed the morphological abnormalities of goat spermatozoa after 48 h and 96 h liquid storage, such as plasma membrane swollen and shrunken, acrosome exfoliation, and axoneme exposure. These ultrastructural changes in liquid-stored goat spermatozoa were similar to the reported observations (30, 52), which indicated that liquid storage induced aberrant structures and impaired quality of goat spermatozoa. Interestingly, a large number of vesicles was found in the plasma membrane of head region and the mitochondria of midpiece region. The appearance of vesicles offers an important speculation about the possible formation of autophagy in liquid-stored goat spermatozoa.

Recently, many studies focus on the association between autophagy and the survival of spermatozoa and have confirmed the existence of autophagy in ejaculated spermatozoa (8, 33, 38). In impaired spermatozoa from cryptorchid patients, in addition to numerous morphological abnormalities, some double-membrane-limited autophagic vesicles were also observed in close proximity to the nuclei and mitochondria, indicating the activation of autophagy in cryptorchid spermatozoa (30). In ejaculated human spermatozoa, several vesicles corresponding to autophagy initiation or autophagosome formation were detected in the head and midpiece regions by TEM analysis (38). Moreover, the formation of autophagy is usually evaluated by a morphological “gold standard” that the observation of double-membrane autophagosome by high-resolution electron microscopy (14, 30). Therefore, in the present study, the ultrastructural changes of goat spermatozoa were further detected by TEM analysis under liquid storage. Notably, massive autophagy vesicles with double-membrane structure were obviously observed both in the aberrant head and tail regions, which verifies the supposition from SEM observation. More importantly, several vesicles were found to wrap some cytoplasmic components even mitochondria, preparing to degradation. These morphological observations are consistent with previous studies (30, 38), and conclude that autophagy is triggered by liquid storage in goat spermatozoa.

The process of autophagy is a lysosome-mediated pathway and regulated by a series of ATG proteins, which have been also considered as molecular markers to assess the progression of autophagy (10, 13, 15). Numerous studies have demonstrated the expressions of critical autophagy-regulating proteins in spermatozoa from various species (34, 39, 53). In this study, to further validate the occurrence of autophagy in liquid-stored goat spermatozoa, the LC3, ATG5, and p62 proteins were selected to analyze their immunolocalization and expression levels after liquid storage. The processed LC3 protein, namely the form of LC3-II, mediates the formation of autophagosome and the membrane expansion and has been widely used to study cellular autophagy levels (16, 17). Studies in stallion spermatozoa found that the conversion of LC3-I to LC3-II significantly increased during cryopreservation and revealed that autophagy may play roles as a pro-survival mechanism in the survival of spermatozoa (34, 36). Our results also showed a significant increase in the ratio of LC3-II / LC3-I by western blot analysis and an enhanced immunofluorescence in LC3 protein after liquid storage, which indicate the increase in autophagy levels in goat spermatozoa. ATG5 has been reported to participate in a ubiquitin-like conjugation system of ATG12-ATG5 that is related to the formation of autophagosome and essential for autophagy (19, 20). Previous studies showed that ATG5 play multiple roles in the formation and function of spermatozoa, such as nuclear shape formation and acrosome formation (32). In the present study, the immunofluorescence of ATG5 was mainly observed in the head of spermatozoa, which may be associated with the putative functions of ATG5 in goat spermatozoa. Moreover, the up-regulated expression of ATG5 protein was detected at 48 h and 96 h after liquid storage, which in combination with the similar expression tendency of LC3-II strongly supports the activation of autophagy in liquid-stored goat spermatozoa. In addition, functional researches have highlighted that p62 has roles in facilitating protein degradation through autophagy, and it is induced to degrade by autophagy (21, 54). The alteration of p62 levels may affect the autophagic flux (22, 55). As expected, contrary to the expression patterns of LC3-II and ATG5, the expression of p62 protein was significantly decreased at 48 h and 96 h after liquid storage, which further validated the autophagy of goat spermatozoa.

The principal roles of autophagy are considered as a cellular stress response (50). The probable contribution of autophagy to promote the survival or death of spermatozoa is still indefinite under *in vitro* preservation (8, 33). Aparicio et al. (38) reported that autophagy was functionally activated in ejaculated human spermatozoa and may have a positive role in human sperm physiology. Uribe et al. (33) reported that the formation of autophagy was detected under stressful conditions, and autophagy inhibition induced a significant decrease in the motility and quality of spermatozoa. Nevertheless, another study found that adding autophagy inhibitors to the extenders during cryopreservation improved the viability of spermatozoa (36). In the current study, the morphological and molecular evidences suggested that autophagy of goat spermatozoa occurred under liquid storage and was accompanied by the aberrant structures and impaired functions of spermatozoa. To clarify the function of autophagy in spermatozoa, chloroquine was used to inhibit autophagy of spermatozoa, and then the effects on spermatozoa quality parameters were investigated. Chloroquine is a well-known autophagy inhibitor and functions in blocking autophagosome-lysosome fusion and the subsequent degradation (56, 57). It is reported that the addition of chloroquine resulted in the accumulation of LC3-II and p62 proteins and obviously suppressed the proceeding of autophagy (33, 38, 57). Similarly, using chloroquine to treat the liquid-stored goat spermatozoa, the increases in LC3-II and p62 protein levels were detected in our studies, suggesting the inhibition of autophagy in goat spermatozoa. Moreover, the motility, viability, plasma membrane integrity, and acrosome integrity of goat spermatozoa were significantly decreased after chloroquine-induced autophagy inhibition. The decreases in the vital quality parameters suggest the important roles of autophagy in spermatozoa under liquid storage. In addition, the significant changes in the levels of ROS, GSH-Px and MDA in chloroquine-treated spermatozoa were also found by being compared to the untreated control. Furthermore, a major challenge for semen liquid storage is to maintain the capacitation of spermatozoa (42, 43). It has been well documented that the alterations in Ca^2+^ influx and protein tyrosine phosphorylation of spermatozoa appear to be correlated to the capacitation of spermatozoa (42, 43). In our study, the significant decreases in Ca^2+^ influx and tyrosine phosphorylation of spermatozoa that indicated the decreases in the function of spermatozoa, were found after chloroquine-induced autophagy inhibition, which further demonstrated that autophagy may be required for maintaining the quality and capacitation of spermatozoa under liquid storage. Overall, these results indicate that autophagy may play a protective role in goat spermatozoa under liquid storage, which provides insights into the future studies on the underlying mechanisms of autophagy regulating the survival of spermatozoa.

## 5 Conclusion

In this study, the occurrence of autophagy in liquid-stored goat spermatozoa was validated by morphological analysis and expression analysis of critical proteins. SEM analysis showed the special typical morphologic abnormalities of spermatozoa after liquid storage and found a large number of vesicles around the plasma membrane of head and the mitochondria of spermatozoa, which firstly implies the formation of autophagy in goat spermatozoa. Further TEM analysis observed massive autophagy vesicles with double-membrane structure around the aberrant spermatozoa, which supports the activation of autophagy in spermatozoa under liquid storage. The expression changes in autophagy-related proteins also validated the occurrence of autophagy. Moreover, chloroquine-mediated autophagy inhibition significantly reduced the quality of spermatozoa. The decreases in Ca^2+^ influx and protein tyrosine phosphorylation of spermatozoa after chloroquine treatment indicated the decrease in fertility of spermatozoa. These results indicate the protective roles of autophagy in the survival of spermatozoa under liquid storage, which provides references for revealing the mechanisms of autophagy regulating the liquid storage of goat spermatozoa.

## Data Availability

The original contributions presented in the study are included in the article/supplementary material, further inquiries can be directed to the corresponding authors.
